# Proximate cues to phases of movement in a highly dispersive waterfowl, *Anas superciliosa*

**DOI:** 10.1186/s40462-015-0048-3

**Published:** 2015-09-01

**Authors:** John F. McEvoy, David A. Roshier, Raoul F. H. Ribot, Andy T. D. Bennett

**Affiliations:** Centre for Integrative Ecology, Deakin University, Locked Bag 20000, Geelong, VIC 3220 Australia; Zoology, School of Environmental and Rural Science, University of New England, Armidale, NSW 2351 Australia; Australian Wildlife Conservancy, PO Box 6621, Halifax Street, Adelaide, SA 5000 Australia

**Keywords:** Arid zone, Behavioural flexibility, ENSO, Movement ecology, Nomadism, Random forest, Rapid environmental change

## Abstract

**Background:**

Waterfowl can exploit distant ephemeral wetlands in arid environments and provide valuable insights into the response of birds to rapid environmental change, and behavioural flexibility of avian movements. Currently much of our understanding of behavioural flexibility of avian movement comes from studies of migration in seasonally predictable biomes in the northern hemisphere. We used GPS transmitters to track 20 Pacific black duck (*Anas superciliosa*) in arid central Australia. We exploited La Niña conditions that brought extensive flooding, so allowing a rare opportunity to investigate how weather and other environmental factors predict initiation of long distance movement toward freshly flooded habitats. We employed behavioural change point analysis to identify three phases of movement: sedentary, exploratory and long distance oriented movement. We then used random forest models to determine the ability of meteorological and remote sensed landscape variables to predict initiation of these phases.

**Results:**

We found that initiation of exploratory movement phases is influenced by fluctuations in local weather conditions and accumulated rainfall in the landscape. Initiation of long distance movement phases was found to be highly individualistic with minor influence from local weather conditions.

**Conclusions:**

Our study reveals how individuals utilise local conditions to respond to changes in resource distribution at broad scales. Our findings suggest that individual movement decisions of dispersive birds are informed by the integration of multiple weather cues operating at different temporal and spatial scales.

**Electronic supplementary material:**

The online version of this article (doi:10.1186/s40462-015-0048-3) contains supplementary material, which is available to authorized users.

## Background

Movement strategies, including obligate migration, facultative migration and nomadism, can be viewed as the mapping of actions (e.g. initiation of long distance flight) onto cues (e.g. day length or weather conditions) [1]. A successful movement strategy will utilise cues that act as proxies for elements that directly affect an individual’s survival and fitness [1]. Several studies have shown that migratory birds adjust the initiation of long distance flight from staging sites in response to daily weather conditions, including wind, temperature and barometric pressure [2–7]. Weather conditions en route, such as changes in wind speed and direction [8] and air temperature [9], have also been shown to influence the movement behaviour of birds that are already migrating.

Waterfowl in arid climates provide a model system to examine responses to rapid change in resource distributions because they have evolved to respond to the ‘boom and bust’ in resource availability characteristic of these regions [10–12]. By studying species that have closely related members of the same genus in other biomes, where they exhibit different movement behaviours, we can gain insights into the breadth of responses to changes in resource distribution and the cues used to initiate movement. While some migratory species in more predictable seasonal biomes are constrained in terms of their physiology and habitat requirements (e.g. shorebirds) many species are known to display flexibility in their response to local and regional weather and landscape conditions [8, 13–15]. Rather than one set of clearly defined characters homologous among all migratory birds, observed patterns of long distance movement are perhaps better viewed in terms of individualistic responses to fluctuations in resource distributions [15–20] modulated by cognitive [21, 22] and physiological constraints [23–27] . Therefore, the position of a species or individual along any continuum of movement behaviour from advective to dispersive [28], or from migratory to nomadic [29, 30], can be considered subject to change within an individual’s lifetime or even between seasons.

There have been few empirical studies on the environmental cues employed by birds in the less seasonal and less predictable environments, such as is found in the arid regions of the southern hemisphere [10, 31, 32]. Theoretical work has suggested resource distributions can be an important driver of nomadic movement behaviour in landscapes where a high proportion of the available resource patches change their distribution over time [21, 33]. Empirical studies on the shift between nomadic and sedentary behaviour in Eastern grass owl (*Tyto longimembris*) [34] and the movements of snail kites (*Rostrhamus sociabilis*) [35] have demonstrated the link between changing resource distributions and movement decisions of nomadic species.

In this study we sought to determine the relative importance of temporary environmental cues in predicting changes in movement behaviour, specifically the initiation of exploratory behaviour and less frequent long distance oriented movements. We tested two main hypotheses: 1) Initiation of long distance movement in Pacific black duck is preceded by phases of exploratory behaviour; 2) Initiation of, exploratory and long distance oriented movement can be predicted by changes in local meteorological conditions, such as an increase in local rainfall and air temperature. While rainfall has long been recognised as a driver of long distance movement in nomadic waterfowl [36], later research suggested that these responses are individualistic and play out on broad spatial scales to changes in the availability of wetland habitat [10, 37].

## Results

**Fig. 1:**
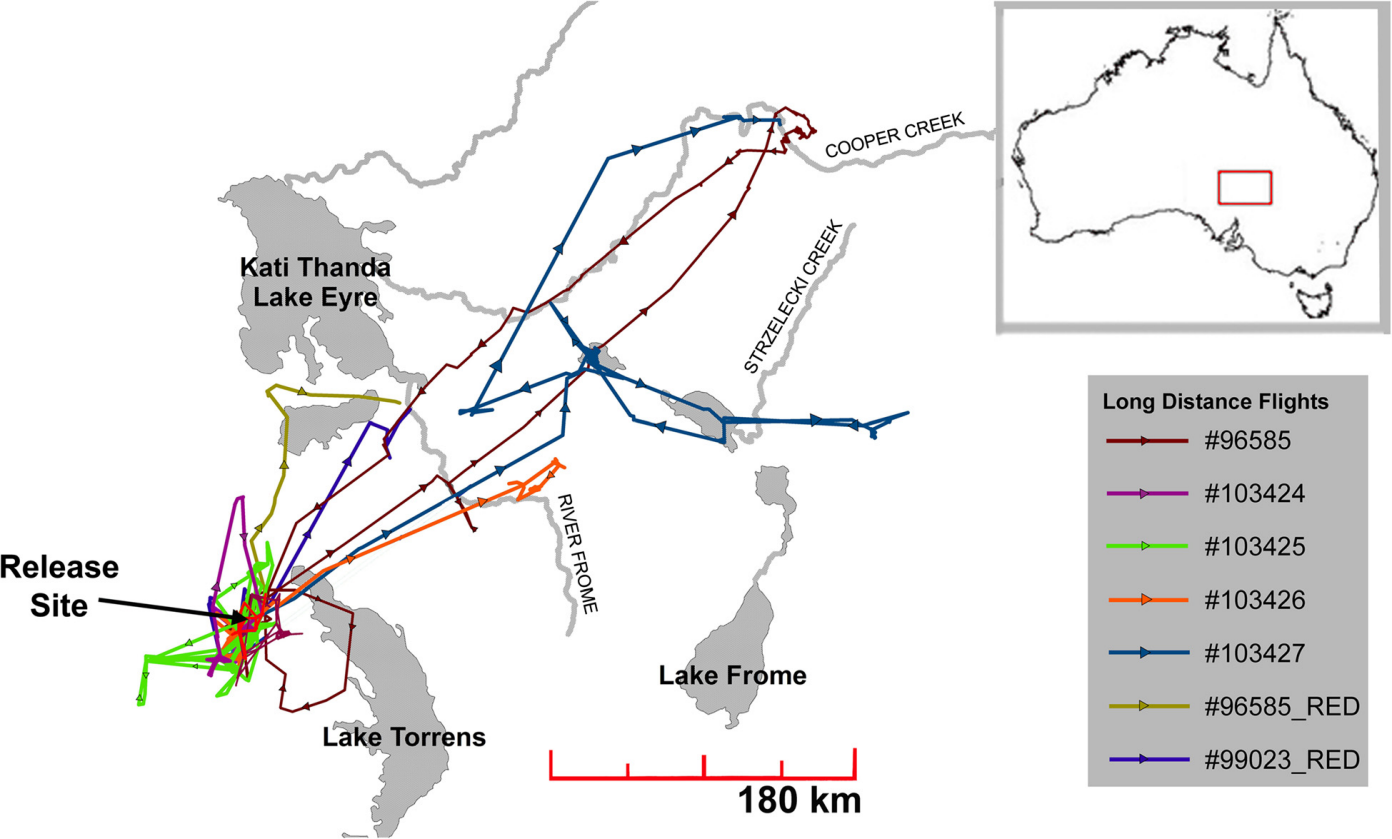
Trajectories of all 20 Pacific black duck released from December 2009 to September 2011. Arrows show direction of travel. Seven individuals undertook long distance movements at various times throughout the study period visiting ephemeral wetlands throughout the region. For all long distance movements taken over the study period only one long distance step (>100 km) occurred during the day; all other long distance movements were nocturnal. Large lakes shown on the map (Kati Thanda-Lake Eyre, Lake Torrens and Lake Frome) are extensive salt lakes that rarely fill and present little or no viable habitat available for Pacific black duck

**Fig. 2:**
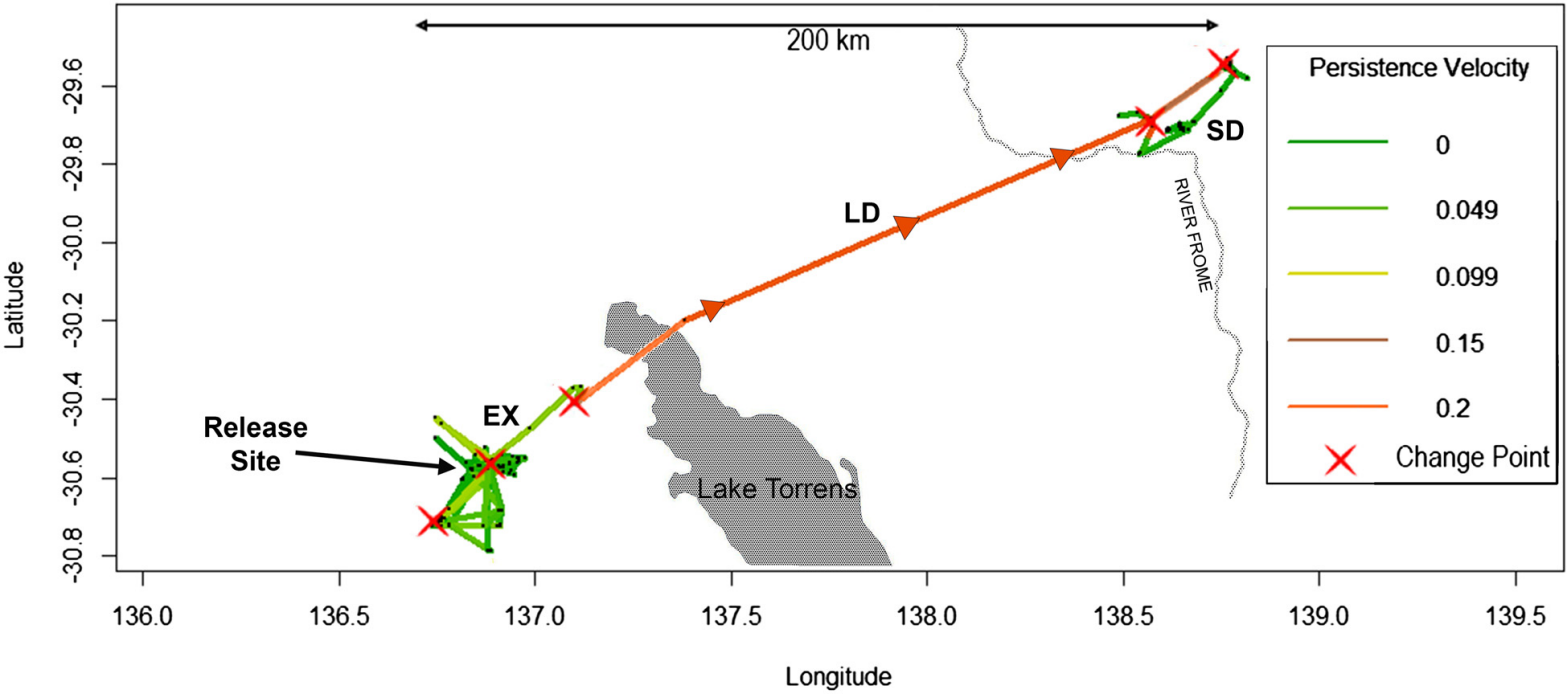
Trajectory of an exemplar individual (#103426) showing behavioural change points along the trajectory (red X’s) and coloured by persistence velocity, the tendency and magnitude of a movement to persist in a given direction. Phases of movement behaviour determined through BCPA are marked (SD = sedentary, EX = exploratory, LD = long distance oriented flight), Change points were detected as this bird initiated exploratory flights around the release site followed by a long distance flight to the north east, crossing the northern tip of Lake Torrens and flying towards a flooded section of the River Frome, where it settled into a sedentary phase

Of the 20 birds tracked using GPS transmitters, for seven individuals BCPA detected a change point that showed a significant increase in mean persistence velocity (indicating faster, more oriented movements). The phases following these change points (*n* = 23 out of 2648 observations) were termed as ‘long distance oriented’ movement. For all seven individuals the detected change point corresponded with an individual initiating long distance movements, moving a distance of >100 km in 24 h at some time during the study period of 22 months (Figs. 1 and 2). Displacement of >100 km occurred at night, except in one instance where an individual undertook a flight of 180 km during the day. In 18 of 20 birds multiple change points (*n* = 76 out of 1991 observations) were detected indicating a shift from a sedentary phase into a phase of movement behaviour distinct from long distance oriented movement or sedentary behaviour, which was labelled as an exploratory phase. Change points at the beginning of exploratory phases showed a significant increase in variability around the mean persistence velocity with no increase in the mean value. Exploratory phases identified by BCPA corresponded with individual trajectories where individuals moved throughout their local landscape on a 30–60 km scale visiting many locations (See Additional file 1: Appendix 1, Figure A2 for examples). Exploratory movement phases represented a large proportion of the overall tracking data for birds in the arid zone (Table 1). Tagged birds spent a mean of 33.9 % of tracking days in these phases of movement with different individuals entering exploratory phases frequently and at different times throughout the study period. Sedentary phases were identified as those showing values of mean persistence velocity and variability close to zero and were the most common phase for individuals (60 % of tracking days; Table 1).

**Table 1:** Total tracking time for each bird, the number of tracking days spent in each phase and days spent in each phase expressed as a percentage of the total

Bird ID	Time Tracked (days)	Sedentary Phases (days)	Exploratory Phases (days)	Direct Flight Phases (days)	%SD	%EX	%LD
96585	29	16	13	0	55	45	0
96585_RED	50	26	18	6	52	36	12
96585_RED_2	77	77	0	0	100	0	0
96586	287	152	119	16	53	41	6
99023_RED	49	25	23	1	51	47	2
103423_RED	207	124	83	0	60	40	0
103421_RED	32	32	0	0	100	0	0
96586_RED	94	82	12	0	87	13	0
99020_RED	90	84	6	0	93	7	0
99035_RED	167	81	86	0	49	51	0
103419	185	50	135	0	27	73	0
103420	19	4	15	0	21	79	0
103421	40	27	13	0	68	33	0
103422	267	178	89	0	67	33	0
103423	69	46	23	0	67	33	0
103424	151	60	89	2	40	59	1
103425	297	219	71	7	74	24	2
103426	205	119	25	61	58	12	30
103427	149	83	33	33	56	22	22
103428	209	148	61	0	71	29	0
Total	2673	1633	914	126	61	34	5

*%SD* percentage of tracking time spent in sedentary phases, *%EX* percentage of tracking time spent in exploratory phases, *%LD* percentage of tracking time spent in long distance oriented flight

**Fig. 3:**
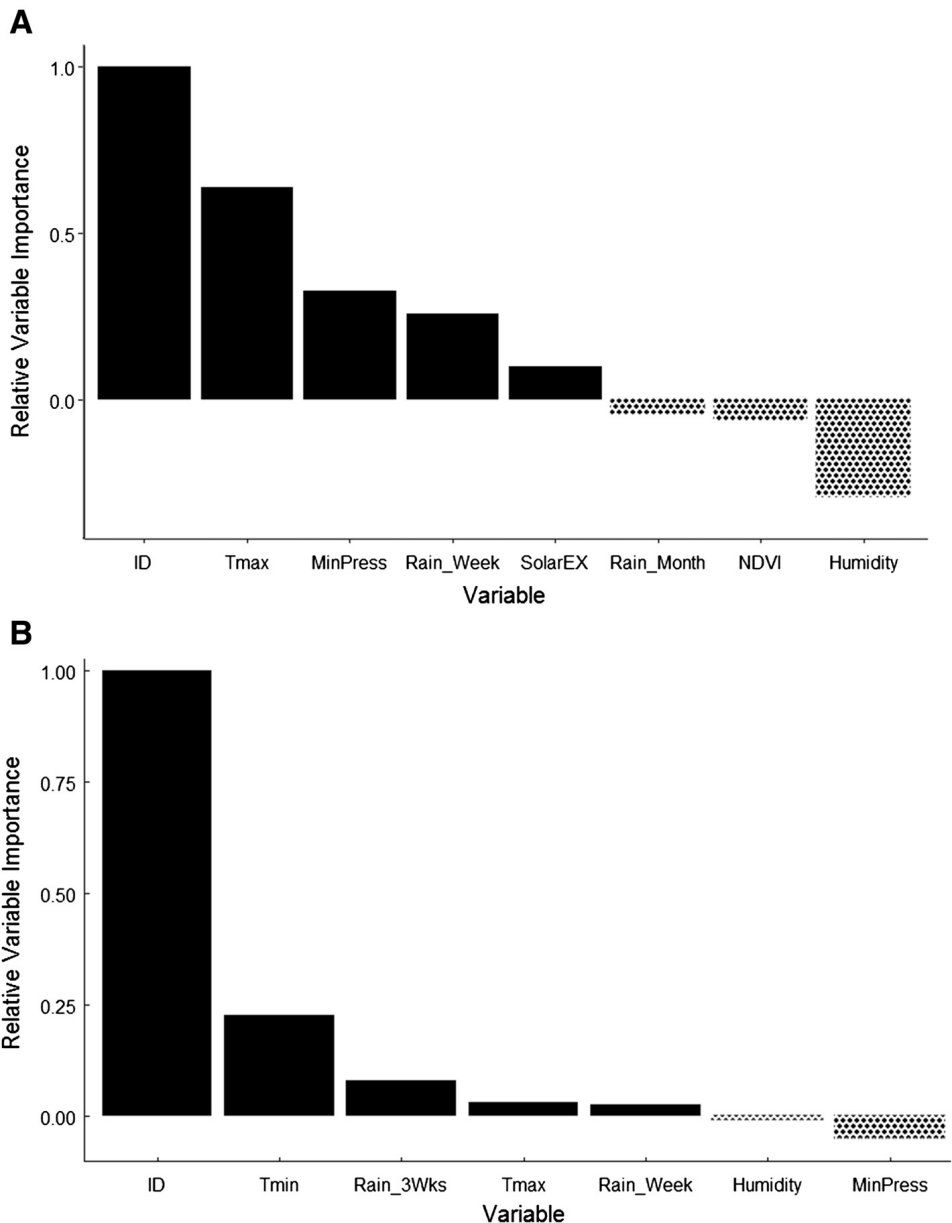
Relative variable importance in the prediction of the initiation of, **a** exploratory behaviour and, **b** long distance oriented flight in Pacific black duck after 2500 iterations of the random forest model. Variables are shown in order of their increasing relevance to increasing the accuracy of predicting a change in behaviour. Variables which had a positive influence on the performance of the model are scaled from 1 to 0 in relation to the most important variable for ease of interpretation. Variables showing negative values are those that, when included in an iteration of the model, reduced the performance of the model. Variables of 0 or negative importance can be ignored as they did not help to increase the predictive power of the model

**Fig. 4:**
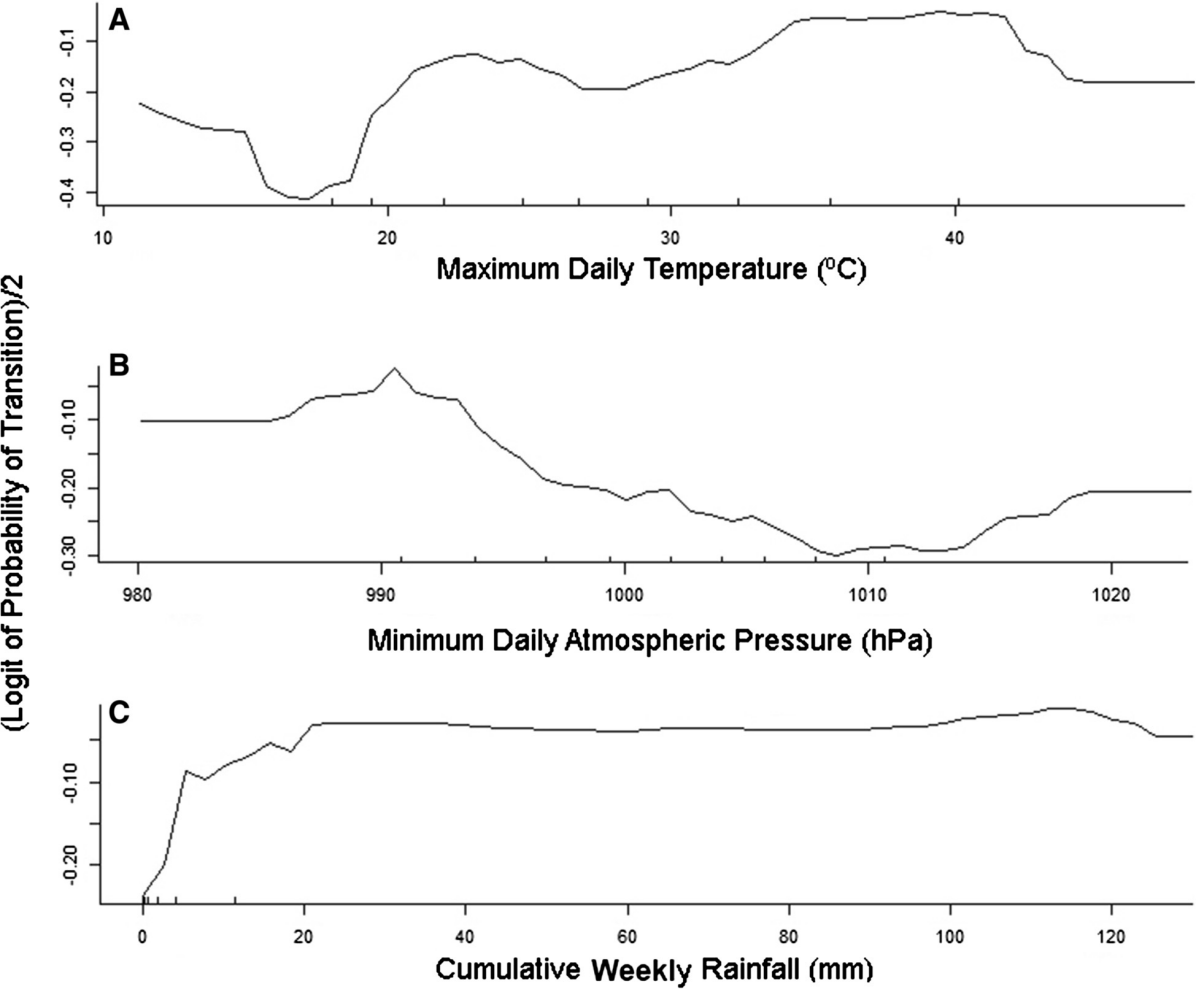
Partial dependence plots for three important variables for predicting initiation of an exploratory phase. **a** Partial dependence on daily maximum temperature. **b** Partial dependence on minimum daily atmospheric pressure. **c** Partial dependence on total weekly rainfall at a bird’s location. Partial dependence is the dependence of the probability of change on one predictor variable after averaging out the effects of the other predictor variables in the model. The Y-axis is half the logit-probability of an individual transitioning to a phase of exploratory behaviour (see Cutler et al. [64] for detailed explanation)

Random forest analysis was used to determine the most important predictors of the initiation of exploratory movement phases. A model which included daily maximum temperature and humidity as well as minimum daily atmospheric pressure, and cumulative rainfall over the past month (Table 2) correctly classified 69 % of cases. Individual bird ID was the highest ranked predictor of the initiation of exploratory behaviour. Daily maximum temperature was the next ranked predictor of initiation of exploratory phases (Fig. 3) with an increasing probability of exploratory behaviour at temperatures above 20 °C and a sharp drop off as temperatures rise above 40 °C (Fig. 4). The third ranked predictor was daily minimum atmospheric pressure (Fig. 3). At low pressure (<990 hPa), the influence of this variable was very low. The influence of minimum daily atmospheric pressure on the probability of initiation of exploratory phases increased at higher pressure levels (Fig. 3). Cumulative local rainfall over the past week was the fourth ranked predictor (Fig. 3) and showed a sharp increase in influence above 5 mm of rainfall after which the influence of this variable remains constant across a range of rainfall values (Fig. 4).

**Table 2:** Accuracy rates for random forest models using initiation of phases of exploratory behaviour (EX) or initiation of long distance oriented flight (LD) as a response and weather/environmental variables as predictors

Response	Set of variables available for each tree	Area under ROC curve—training	Area under ROC curve—validation	% Classification Accuracy—training	% Classification Accuracy—validation
Initiation of exploratory flight	Bird ID	0.98	0.97	74	69
	Tmax				
	MinPress				
	Humidity				
	Rain_Week				
	SolarEx				
	Rain_Month				
	NDVI				
	Humidity				
Initiation of long distance oriented flight	Bird ID	0.92	0.78	71	72
	Tmin				
	Tmax				
	MinPress				
	Humidity				
	Rain_3Wks				
	Rain_Week				

The ROC (Receiver Operating Characteristic) curve is a means of measuring the performance of a binary classifier by plotting true positives against false positives, giving an indication of the sensitivity of the model. For explanation of abbreviations see Table 3 below. See Table A2 in Additional file 1 for examples of models using combinations of variables that did not produce classification accuracies high enough to warrant inclusion in the main results

**Fig. 5:**
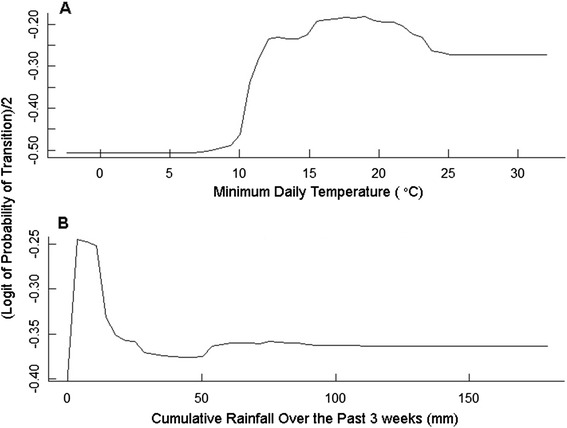
Partial dependence plots for two important variables for predicting initiation of a long distance oriented phase. **a** Partial dependence of the response on minimum daily temperature. **b** Partial dependence of the response on cumulative rainfall over the past three weeks. Partial dependence is the dependence of the probability of change on one predictor variable after averaging out the effects of the other predictor variables in the model. The Y-axis is half the logit-probability of an individual transitioning to a phase of long distance oriented flight (see Cutler et al. [64] for detailed explanation)

In order to investigate the initiation of phases of long distance oriented movement, a random forest model using minimum daily atmospheric pressure, minimum daily temperature and humidity and local rainfall in the past three weeks achieved 72 % accuracy in classifying the rare initiation of long distance oriented movement (Table 2). While the predictor variables in this model are similar to the previous model focusing on exploratory phases, their ranking in terms of their influence on the response differ (Fig. 3). The variable of greatest importance was individual (Fig. 3), indicating that individual flexibility plays a major role in the initiation of long distance oriented movement. Daily minimum temperature was ranked second in order of influence on the initiation of long distance oriented flight and cumulative rainfall in the past three weeks was ranked third, with a relatively small influence. Partial dependence plots for these predictor variables (Fig. 5) show that the probability of initiation of a phase of long distance movement behaviour increases as minimum daily temperature increase with the greatest influence occurring at higher temperatures. Probability of initiation of long distance movement is also influenced by cumulative rainfall, with a rise in probability of initiation when rainfall is at low to medium levels (5–10 mm) and falling off rapidly as the amount of cumulative rainfall increases. The influence of the remaining predictor variables is much smaller in comparison to that of individual bird ID, which is not unexpected given the overall rarity of these long distance oriented phases in the original data set.

## Discussion

Our findings reveal that the movement behaviour of Pacific black duck living in an arid environment can be described by three different movement phases (sedentary, exploratory or long distance oriented flight). Exploratory flights were a common occurrence and preceded all but one case of long distance oriented flights by individuals (22 out of 23 events). The movement behaviour of nomadic species has been previously characterised as a direct response to fluctuating resource distributions with individuals ‘ranging’ through a landscape until a suitable habitat patch is encountered [38, 39] . In recent years a more complex picture of nomadic movement has emerged because nomadic species from a range of taxa have been found to undertake highly oriented movements across long distances to specific locations apparently in response to cues such as vegetation growth [40] regional rainfall [10] and internal factors such as moult schedule [41].

The current study expands on previous work on nomadic waterfowl in arid Australia [10, 42] by using new analyses to quantify a range of movement behaviours and investigating cues for the initiation of movement behaviour rather than looking solely at the outcomes of movement decisions such as the distance moved or habitat type at the destination. The results of random forest analysis (Fig. 3) suggest that the initiation of long distance oriented movement was highly individualistic and influenced by local meteorological conditions, specifically air temperature and rainfall. In arid landscapes where the distribution of resources is patchy and irregular, weather conditions have been shown to be an important cue for bird movement [43, 44]. Studies on migratory birds have shown that timing of departure can be linked to fine scale changes in wind conditions [45] as well as ambient temperature atmospheric pressure [46]. Ours is the first study to demonstrate how similar fine scale changes in local meteorological conditions can be used to predict the movement decisions of individual birds in arid landscapes where resources are distributed patchily in time and space.

What constitutes a movement strategy of an individual can be difficult to define and is influenced by the time window during which an animal is studied. Instead, observed movement patterns should perhaps be considered as individualistic responses to environmental conditions within the species-specific constraints of physiology and spatial memory [17, 22, 25, 28, 47, 48]. Therefore, the position of a species or individual along any continuum of movement behaviour from advective to dispersive [28], or from migratory to nomadic [29, 30], can be considered subject to change within an individual’s lifetime or even between seasons. Over short periods of days or even weeks the Pacific black duck tracked in our study could be said to show a sedentary movement strategy. However, with an extended tracking period we see that while all birds did spend the largest portion of their total time in sedentary phases (Table 1), these periods were punctuated by frequent bouts of exploratory behaviour (in 18 out of 20 birds) after which some individuals made the decision to undertake long distance movement (7 out of 20 birds) and others did not. This suggests that choosing to remain sedentary is just one of the possible choices available to this species, after gathering information through exploratory movements and taking into account physiological conditions and other potential trade-offs as seen in other arid landscape birds [43].

The extensive flooding during our study, which arose from ENSO being in a La Niña phase, occurs rarely and our study provided an infrequent opportunity. With the current data set it was not possible to track the movements of birds during the drier conditions brought by a contrasting El Niño period. An obvious next step for future work is to analyse movements of waterfowl during El Niño conditions, which may produce different movement patterns as individuals are forced out of an area by deteriorating local conditions [43] and are constrained to a smaller range of viable destinations [49]. The Pacific black duck is closely related to migratory species from temperate biomes such as the mallard (*Anas platyrhynchos*) [50, 51] which displays migratory or partial migratory behaviour [15]. Many studies in seasonally predictable environments in the northern hemisphere temperate biomes have related departure dates of migratory birds to changes in variables such as day length and temperature [2], wind conditions [5, 46] and food availability [3]. These studies show departure decisions being influenced by meteorological conditions and local habitat quality but only in a limited timeframe, within which all individuals are expected to depart. In this study we have shown that a closely related species uses similar cues to move between patchily distributed resources for which there are no reliable seasonal cues to indicate their presence. The individualistic nature of movement decisions shown in this study is comparable to those of Oppel et al. [14], who demonstrated that migratory king eiders (*Somateria spectabilis*) in the Northern Hemisphere are also highly individualistic in their movement decisions and utilise environmental cues, such as the concentration of sea ice in their locality for the initiation of exploratory behaviour to inform future movement decisions.

### Exploratory movement phases

While initiation of exploratory phases of movement was more common in the data set than initiation of long distance movement, they remain a comparatively rare event. There may be a number of motivations for engaging in this type of movement, for example foraging, searching for mates, sampling the landscape (prospecting) or escaping predators, but we cannot address these with the current data set. The classification accuracy of the random forest model compares favourably with studies using machine learning to classify events in biophysical systems [52, 53].

In the model, the highest ranking predictor of initiation of exploratory behaviour was individual bird ID. As with long distance behaviour this indicates highly individualised movement behaviour with different birds choosing to explore their environment at different times. It should be noted that not all birds were in the same location at the same time, and hence experienced different environmental conditions.

The partial dependence plots (Fig. 4) indicate that birds are more likely to explore when low pressure systems pass through the area bringing small amounts of rain (>5 mm). The influence of atmospheric pressure on the response is lowest under conditions of high pressure (>1012 hPa) and high temperature (>40 °C), associated with dry conditions and low wind speeds. This may reflect the constraint of maintaining water balance while undertaking flights in arid landscapes [54]. Taken together, these findings suggest that arid zone waterfowl can take advantage of even small pulses in rainfall brought by passing rain systems to explore their local habitat while conditions are good, and are less likely to invest in exploratory behaviour during drought conditions.

The observable cues in a bird’s local environment may be used as a proxy for distant conditions at an eventual destination [10] but may also be important cues for preparatory behaviour such as accumulating energy reserves or undergoing moult [1]. Our findings show that, in an environment where the distribution of resources can change rapidly, birds utilise changes in daily local meteorological conditions as proximate cues for the initiation of exploratory behaviour, as the constraints of habitat availability are decreased. Similar studies [35] have suggested that nomadic birds may explore more in times of resource abundance to familiarise themselves with high quality habitat patches, decreasing the need for fruitless searching in the future. Similar results were observed in seasonally breeding trumpeter hornbills (*Bycanistes bucinator*) by Lenz et al. [55], which engaged nomadic behaviour when not constrained to central place foraging by breeding. Our findings suggest that engaging in exploratory behaviour in response to changes in their local habitat may be a key process in the movement decisions of many other closely related species.

### Long distance movement phases

BCPA analysis revealed a pattern of repeated phases of exploratory behaviour preceding phases of long distance oriented movement, suggesting that Pacific black duck are capable of integrating information from their immediate vicinity, with information of conditions in the broader landscape when making decisions about movement. The random forest model was able to correctly classify initiations of long distance oriented flight in 72 % of cases, which compares favourably to a similar study on the movement decisions of wintering sea ducks in the Arctic [14]. The small number of individuals which actually undertook long distance oriented flight (7 out of 20) lead to individual ID being ranked as the strongest predictor.

Beyond the strong influence of individual variation, as observed in studies on related species in this landscape [42], the initiation of long distance movement is found to be associated with daily minimum temperature at a bird’s location. As daily minimum temperature rises above 10 °C the probability of initiation increases (Fig. 5). Given that long distance flight in this study occurred almost exclusively at night this result suggests that birds are reluctant to undertake long distance flights on colder nights.

At the same time, cumulative rainfall over the previous three weeks at a bird’s location shows a similar pattern of influence with low levels of cumulative rainfall (and hence less aquatic habitat available in this arid environment) having little influence. Once cumulative rainfall rises above 5–10 mm we see the influence of this variable increase sharply and then drop again at higher levels of local rainfall (Fig. 5). This pattern of influence may indicate the boundaries within which Pacific black duck will undertake movement away from a given area. If the landscape is dry with cumulative rainfall close to 0 mm, they choose to remain on small permanent water bodies such as sewage treatment works. With even a small increase in cumulative rainfall, and hence an increase in natural habitat available in the landscape, they are more likely to undertake a long distance journey. If their local landscape has received high levels of rainfall and is flooded they may choose to remain in the area and exploit the boom of resources this would provide.

The individualistic nature of the response to meteorological cues in Pacific black duck reflects similar findings from studies on nomadic waterfowl in arid landscapes [24, 41, 42]. Although only external factors were quantified in this study, we suggest that this highly individualistic response may be influenced by internal physiological cues and ecological factors, such as energy reserves, breeding and predation risk as suggested by other studies [17, 56] but which were beyond the scope of the current study. In future studies sophisticated biotelemetry methods [57] could be deployed to provide information on the physiological state of nomadic birds as they undertake long distance flights.

The meteorological predictors of the initiation of long distance movements identified in the present study may not necessarily be the proximate drivers of movement. The changes in meteorological conditions may correlate with some unknown environmental covariate that individual birds respond to directly. Future work could explore other causes of variation in the movement patterns of arid zone waterfowl such as reproductive state, age and body condition and the use of biotelemetry methods [57] to gain insights into the physiological state of individual birds as they undertake long distance journeys.

In the present study we focus solely on the initiation of movement phases, not on their cessation. While an investigation of the factors contributing to an individual’s decision to stop at a given location (e.g. spatial memory, presence of conspecifics, abundance of food, physiological state) would be of great interest, this was beyond the scope of the current study.

## Conclusions

Our findings reveal that, in Pacific black duck, the decision to initiate exploratory movements and long distance flights is a highly individualistic process that can be predicted by local weather conditions. Our study species is a member of a globally distributed genus which includes migratory, partially migratory and sedentary species. These findings begin to reveal how birds respond to weather variability and provide insight into how individuals respond to changes in resource distribution at broad scales. The demonstrated response to short term weather conditions, the individualistic nature of that response and the use of exploratory movements show that waterfowl are capable of fine tuning their movement strategies, drawing upon information from a range of different environmental cues. Understanding which taxa can similarly respond to variable patterns of resource availability will aid conservation efforts as weather patterns intensify or change frequency as global climate patterns change.

## Methods

### Study species

The Pacific black duck (*Anas superciliosa*) is a widely distributed dabbling duck with a range covering much of Australia and extending into New Guinea, Indonesia and New Zealand [58]. The species is commonly observed on farm dams and man-made infrastructures such as municipal ponds and sewage works as well as on remote ephemeral wetlands [12, 58–60]. The Pacific black duck is one of several Australian representatives of the globally distributed genus *Anas* that have closely related species occupying markedly different habitats in other biomes.

While Pacific black duck are considered to be dispersive with long periods of sedentary behaviour [58, 59], information on their movements is limited to counts of bird densities and a small number of banding studies. Banding recoveries from different studies around Australia have shown that a small proportion of banded birds show dispersive movements from 100 km to >400 km from their point of release with no evidence of a return to their point of origin, while the majority of birds are thought to remain largely sedentary [61]. However, with banding recoveries there is no way to tell if an individual travelled widely between recoveries and then returned to a favoured location, as shown in grey teal (*Anas gracilis*) [31]. The limited information available from bird counts and banding studies suggests that Pacific black ducks are highly flexible in their movement ecology [36, 59]. As this species appears to display a phenotypic plasticity in its movement behaviour (with populations in different regions of Australia showing different movement patterns [36]) and is known to utilise small ephemeral water bodies as well as large permanent swamps it is an ideal study species to address the issue of individual behavioural flexibility in response to environmental change in an unpredictable landscape.

### Study site and arid inland Australia

Inland Australia is dominated by arid ecosystems (<200 mm average annual rainfall) in which productivity is driven by infrequent and largely unpredictable [28] rainfall and flooding events [62] and animals must respond to fluctuations in conditions driven by long term cycles of ‘boom and bust’ [63, 64]. During the period of this study (2010 and 2011), inland Australia experienced two of the wettest years since the mid-1970s [65] with heavy La Niña rains. For example, at our study site in the region of the Arcoona lake system, South Australia (30.56°S, 136.88°E), sporadic rainfall caused localised flooding over the course of the study, but two major rainfall events occurred that brought regional (and indeed national) flooding. On 9^th^ April 2010, the study area received 86 mm of rainfall, which is more than half the annual average (151 mm) in a single day. More extreme rainfall was caused by the extension of Cyclone Yasi over Central Australia on 6^th^ February 2011, when 96 mm of rain fell over three days; much of the state of Queensland was also flooded by this same cyclone.

A municipal sewage works in a town surrounded by the Arcoona lake system was a focal point for waterfowl activity (Fig. 1) and is one of a small number of permanent man-made water bodies used by waterfowl in the area. All birds were captured in the vicinity of this sewage works. The Arcoona lake system which extends 85 km to the south of the trapping location is the only significant wetland system in the region for waterbirds [66]. This system is composed of 10 large semi-permanent lakes, which contained water for the duration of this study, and numerous swamps and clay pans fed by runoff and rainfall. Cooper Creek to the north and north-east of the study site is an extensive dryland river system that terminates below sea level at Kati Thanda-Lake Eyre, a large salt of approximately 9000 km^2^. This region has the lowest average annual rainfall in Australia; although during the study Cooper Creek was flooded along its entire 1300 km length.

### Tracking using GPS transmitters

Twenty Pacific black duck were caught using mist nets or trapped using baited funnel traps [67]. Thirty gram solar powered GPS transmitters (Microwave Telemetry) were attached using a harness design following Roshier and Asmus [68]. Between December 2009 and October 2011, GPS fixes were collected continuously every two hours throughout the day and night. Displacement over a two hour period was calculated from GPS locations with a nominal accuracy of 15 m. Day and night-time movements were differentiated based on times of first and last light, calculated using civil twilight tables from Geoscience Australia and GPS position of the individual.

### Behavioural change point analysis

Behavioural Change Point Analysis (BCPA), following Gurarie et al. [69] and Garstang et al. [70] was used to detect changes in the movement behaviour of individual birds. BCPA uses velocity, the angle of the trajectories connecting successive GPS points and distance moved between fixes to produce a variable called *persistence velocity*, which represents the magnitude and tendency of a movement to persist in a given direction. Three phases of movement behaviour were determined from the results of BCPA based on the mean value of persistence velocity, the variation around the mean and the degree of autocorrelation. These phases were characterised as Sedentary (SD), Exploratory (EX) and Long Distance Oriented movement (LD). For a detailed description of BCPA and the characterisation of each of these phases, see Gurarie et al. [69], Garstang et al. [70] and Additional file 1: Appendix 1. BCPA analysis was carried out in the R programming environment [71]. Oriented movement is used in this study, as in others [e.g.: 40, 72], to describe a rapid movement with a non-random orientation, between successive GPS fixes, the individual apparently moving toward a known destination [73]. While the movement pattern may appear to an observer as a journey toward a specific destination, this pattern may be the result of following an environmental gradient with no knowledge of the eventual destination.

### Weather and remotely sensed landscape variables

The National Centres for Environmental Prediction (NCEP) Reanalysis II data set [74, 75] was used to gather values for the following weather variables: atmospheric pressure, surface humidity, and surface air temperature at each GPS location (Table 3). Measurements of surface conditions including rainfall and solar exposure (as a proxy for cloud cover), were gathered from daily, weekly and monthly weather data supplied by the Bureau of Meteorology Australia (BOM; Table 3). Measurements of Normalised Difference Vegetation Index (NDVI) were also gathered from the BOM database as a proxy for habitat availability and productivity (Table 3). The surface of small to medium sized water bodies (i.e. diameter ~1 km) in this region are often rapidly covered with emergent vegetation and NDVI is a coarse proxy of wetland availability because wetland extent can change by orders of magnitude over relatively short periods (weeks) [49] and the spectral signature changes equally rapidly [76]. Weather and remote sensing data from all sources were annotated to the tracks of individual birds using the package ‘RNCEP’ [77] in the R programming environment.

**Table 3:** Predictor variables used in random forest models showing the spatial scales at which each variable was analysed

Predictor Variable	Abbreviation	Spatial Resolution
Maximum Daily Relative Humidity (%)	Humidity	0.3^0^ (~33 km)
Minimum Daily Atmospheric Pressure (Pa)	MinPress	0.3^0^ (~33 km)
Maximum Daily Solar Exposure (MJ/m^2^)	SolarEx	0.05^0^ (~5 km)
Maximum Daily Temperature (°C)	Tmax	0.05^0^ (~5 km)
Minimum Daily Temperature (°C)	Tmin	0.05^0^ (~5 km)
Local Rainfall in Past 7 days (mm)	Rain_Week	0.05^0^ (~5 km)
Local Rainfall in Past 3 Weeks (mm)	Rain_3Wks	0.05^0^ (~5 km)
Local Monthly Rainfall (mm)	Rain_Month	0.05^0^ (~5 km)
NDVI Monthly (index 0–1)	NDVI	0.05^0^ (~5 km)

### Random forest analysis

Using data from all 20 individuals, initiation of an exploratory phase was treated as a binary response variable. Environmental variables were only available on a daily basis; therefore the behavioural response was also aggregated to a daily temporal scale. A day where no change in behaviour occurred was assigned a value of ‘0’ and a day where a phase of exploratory behaviour was initiated was assigned a value of ‘1’ (76 out of 1991). Days on which an individual was already in an exploratory phase were excluded from the analysis. A separate analysis was conducted to investigate the initiation of long distance oriented movement. Any day where no change in behaviour occurred was assigned a value of ‘0’ and any day where a phase of long distance oriented movement was initiated was assigned a value of ‘1’ (23 out of 2648). Days on which an individual was already in a phase of long distance oriented movement were excluded from the analysis.

Machine learning methods have found wide application in the analysis of rare events particularly in remote sensing of environmental change at the landscape scale [78, 79]. Machine learning has also been successfully applied to the movement behaviour of marine mammals [52, 80] and waterfowl in the northern hemisphere [14].

As long distance movements of Pacific black duck in this arid environment were infrequent in the data set the binary predictor produced was heavily unbalanced. In order to cope with this unbalanced data set we adopted machine learning methods of classification [81, 82]. Random forest analysis fits many classification trees to the data and provides estimates of variable importance and classification error rate averaged across many random trees [83]. We constructed 2500 classification trees from a subsample of the data set (75 % of observations) used to train the model. Accuracy of prediction was tested against the remaining data. In the creation of each classification tree in the RF model a random subset of predictor variables (4 predictors) was used at each split in the tree, further reducing the ratio between predictors and observations. The overall performance of the model is given by the area under the Receiver Operational Characteristic (ROC) curve; a plot of the rate of false positives against true positives [84]. Variable importance is measured by the mean decrease in accuracy of the model if that variable is removed over repeated permutations. One major strength of machine learning methods such as classification trees (and by extension random forests) is that they do not suffer from pseudo replication in the same way as standard statistical methods as it is not necessary to assume that each data point is independent [81, 83, 85]. By carrying out repeated random sub sampling of the data and the inclusion of ID as a predictor this analysis can handle large datasets with repeated measures from a small number of individuals.

Given the rarity of the response, a model that simply classifies every observation as the majority class (no change in behaviour) would have a high performance (area under the ROC curve) but would fail to predict the rare event of interest. For this reason, we minimised the error in predicting the minority class by adopting a method of “down sampling” [85–87] whereby a random subsample of the majority class (no change in behaviour) is analysed in each classification tree, bringing it into balance with the minority class (change in behaviour). Random forest modelling was carried out using the software package “randomForest” [88] in the R programming environment.

## Additional file

Additional file 1: **Supplementary Material.**
