# Supplementary material for: Proximate cues to phases of movement in a highly dispersive waterfowl, Anas superciliosa
Source: Mov Ecol. 2015 Sep 1;3(1):21. doi: 10.1186/s40462-015-0048-3 (PMC4556217; doi:10.1186/s40462-015-0048-3)
Supplement: Supplementary file 1 — Supplementary Material. [file 40462_2015_48_MOESM1_ESM.pdf]

Supplementary Material

McEvoy, J.F., Roshier, D.A., Ribot, R.F.H., and Bennett, A.T.D., “Proximate cues to phases of movement in a highly dispersive waterfowl, *Anas superciliosa*”

Movement Ecology 000: 000 – 000

Appendix 1

Behavioural Change Point Analysis

Behavioural Change Point Analysis (BCPA), following Gurarie et al [40], was used to detect transitions in the movement behaviour of individual birds. BCPA uses velocity, the angle of the trajectories connecting successive GPS points, distance moved between GPS fixes (step length) and produces a variable called *persistence velocity* (velocity x Cos(turning angle)) which represents the magnitude and tendency of a movement to persist in a given direction. A moving window is scanned along the time series of animal locations and maximum likelihood methods are used to detect significant change points in the characteristics of the movement behaviour. Change points are detected by selecting the most likely model that explains the changes in three properties of the time series of V_p (mean, variance and autocorrelation) between the current window and the previous window. At each change point the model chosen to make the distinction is provided. There are 8 potential models which can be chosen, M0-M7. M0 represents the null model where there is no change in any of the three parameters. M1, M2 and M3 represent one parameter changing significantly while the other two parameters remain constant; M4, M5 and M6 have one equality each while two other parameters change and M7 is the most alternate hypothesis, in which all parameter values change at the change point. A moving window covering 24 hours of movement was used to match the temporal scale at which weather data was collected.

BCPA analysis was carried out in the R programming environment [41].

The significant changes in behaviour that mark the beginning and end of each phase were detected automatically by BCPA. The subsequent characterisation of these phases was carried out in two ways. Firstly by looking at the model that BCPA chose at the change point that begins the phase. At all change points either M2 or M4 was selected as the most likely model. If M2 is selected this represents a change in the variance around the mean but no concurrent change in either the mean value or the autocorrelation value, if M4 is selected this indicates that both the mean value and variance have changed but the autocorrelation value has not. BCPA also provides an indication of the direction of the changes in these parameters. Phase that showed a significant increase in variance and either no change or a decrease in mean V_p were marked as 'Exploratory". Movement phases that have low persistence velocity, increased variance and low autocorrelation represent movements that are highly variable in speed and duration of each step and include many changes of direction. This characterisation of the phases was confirmed by visually inspecting the characteristics of the BCPA graph within each phase for which M2 and M4 were selected at the beginning of the phase.

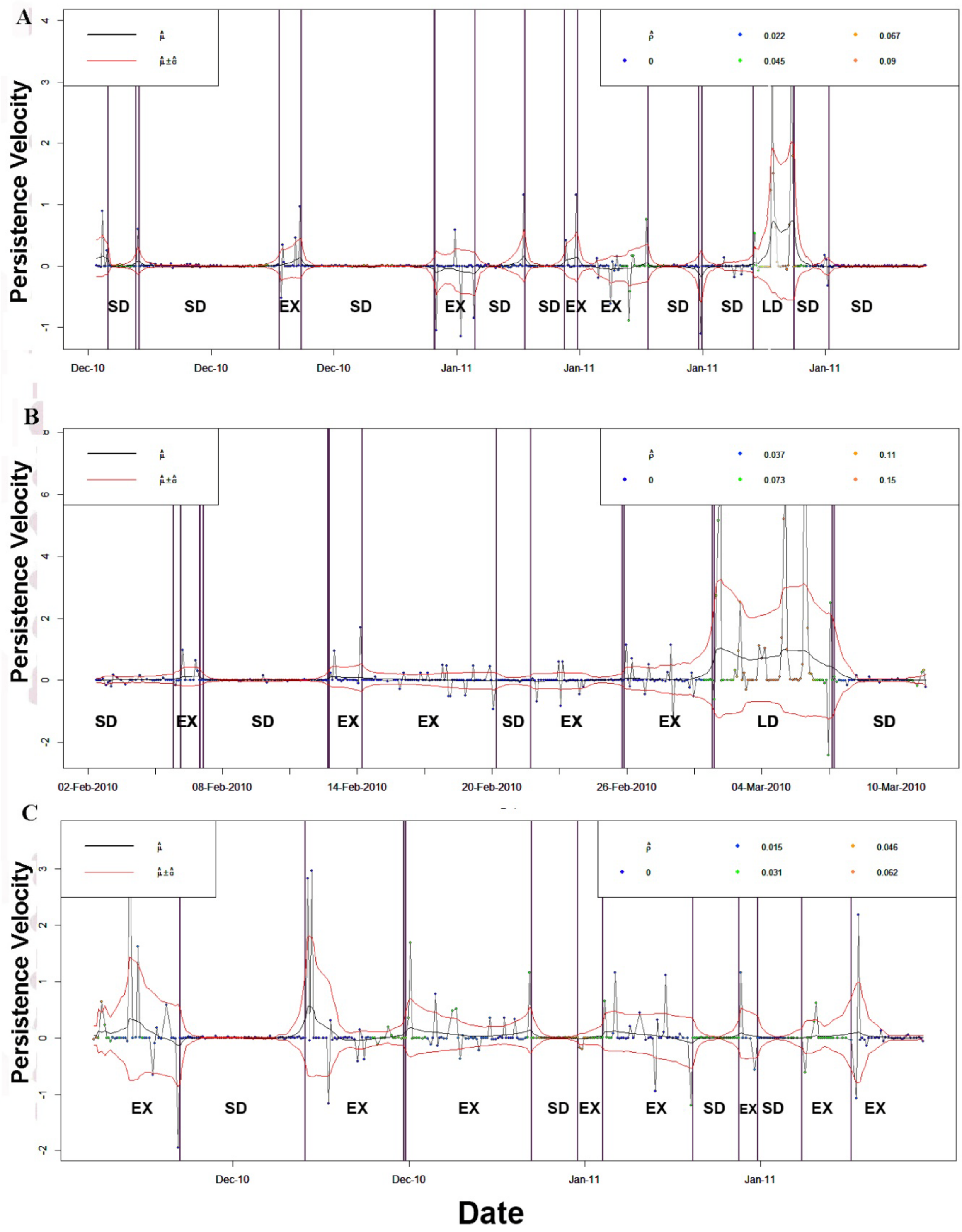

Figure A1: Three representative examples of Behavioural Change Point Analysis (BCPA) for three individual Pacific black duck. Phases of movement behaviour are labelled as SD (Sedentary), EX (exploratory) and LD oriented long distance movement. The Y axis shows

‘persistence velocity’ or the magnitude and tendency of a movement to persist in a given direction. The X axis shows the date. The black line shows changes in the mean value of persistence velocity ($\hat{\mu}$) along the time series an increase in which corresponds to faster and more oriented movement. Red lines indicate variability around the mean ($\hat{\sigma}$) an increase in which indicates a more variable, undirected movement pattern. The points in the time series are coloured according to the measure of autocorrelation between points (ρ). Highly oriented movements show a greater degree of autocorrelation than meandering undirected movement. Purple vertical bars represent points where significant changes in movement behaviour were detected by BCPA. A) During the period shown this individual transitioned from a SD phase to an EX phase followed by a LD phase in mid-January 2011, before settling back to a prolonged SD phase. B) During the period shown this individual displayed a number of EX phases leading up to a brief LD phase in early March 2010. C) During the period shown this individual displayed numerous SD and EX phases and no LD phases were identified.

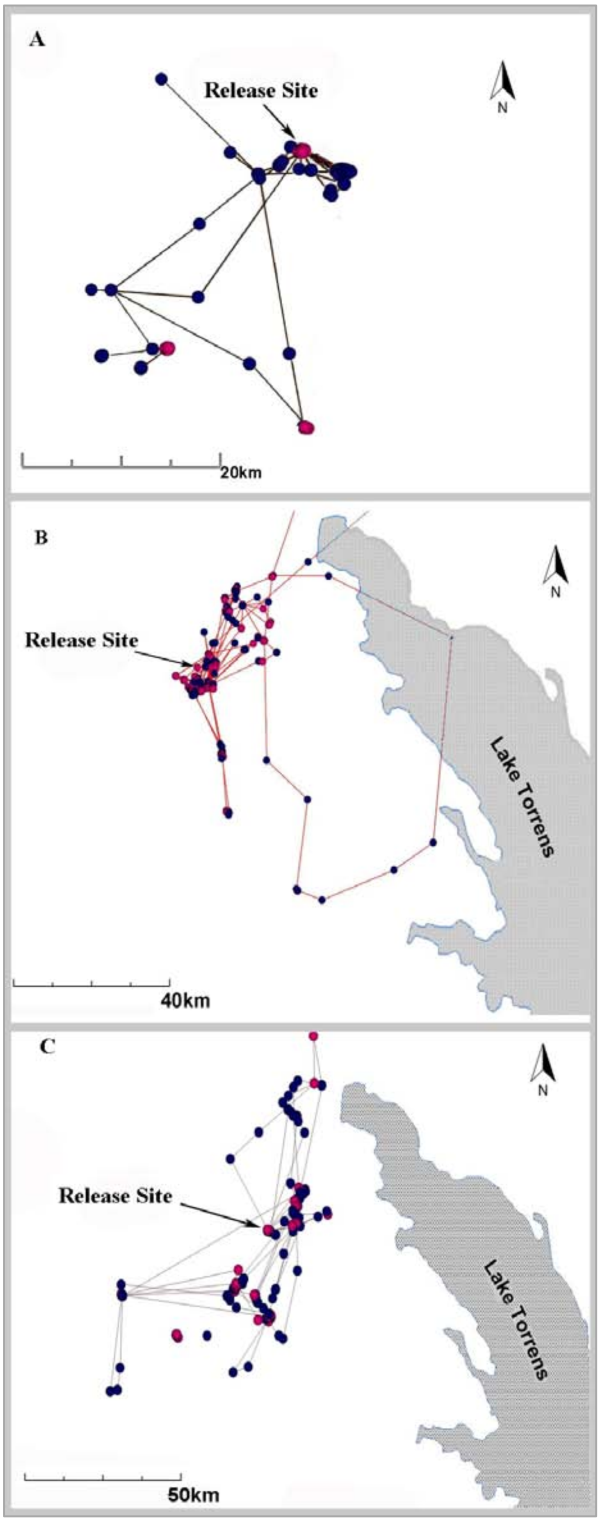

Figure A2: Sections of individual trajectories are taken from an exploratory phase of movement behaviour as determined using BCPA. GPS fixes taken at night are marked in blue; fixes taken during the day are marked in pink. Individuals can be seen exploring the local landscape on a 20-60 km scale. The vast majority of exploratory behaviour takes place

at night. The large lake shown is the northern portion of Lake Torrens an extensive, dry salt lake with no viable habitat at the time.

Table A1: Summary of tracking data for all birds tracked during the study. N/A indicates either no in-flight records for that individual or the transmitter used (22g GPS) was not capable of recording altitude.

ID	Maximum Daily Displacement (km)	Mean Daily Displacement (km)	Maximum Distance From The Origin (km)	Maximum Speed (km/h)	Maximum Altitude (m)	No. Days Tracked
**96585**	18	2	26	39	63	29
**96586**	464	8	468	78	40	287
**103419**	25	1	34	78	73	185
**103420**	1	0	1	80	17	19
**103421**	14	2	22	68	25	40
**103422**	40	2	46	79	65	267
**103423**	59	4	64	87	57	69
**103424**	78	2	89	88	N/A	151
**103425**	159	4	183	50	N/A	297
**103426**	187	2	216	56	N/A	205
**103427**	246	7	461	83	N/A	149
**103428**	22	1	26	56	N/A	209
**103421RED**	1	1	1	2	18	32
**103423RED**	56	3	36	71	25	207
**96585RED**	77	5	177	77	17	50
**96585RED_2**	1	0	1	72	31	77
**96586RED**	9	1	19	80	22	94
**99020RED**	170	2	164	68	35	90
**99023RED**	140	9	178	2	35	49
**99035RED**	25	2	26	79	57	167

Table A2: Examples of random forest models using different susbsets of the total list of predictor variables available. These models did not perform well and produced classification accuracies close to 50% as would be expected by random chance.

Response	Set of variables available for each tree	Area under ROC curve – training	Area under ROC curve – validation	% Classification Accuracy - training	% Classification Accuracy - validation
**Initiation of exploratory flight**	Bird ID<br>Tmin<br>Rain_3Wks<br>Rain_Week	0.71	0.72	42	39
**Initiation of exploratory flight**	Bird ID<br>Tmin<br>Rain_3Wks<br>MinPress<br>NDVI	0.72	0.73	42	35
**Initiation of long distance oriented flight**	Bird ID<br>Tmin<br>Tmax<br>Humidity<br>Rain_3Wks<br>Rain_Week	0.89	0.68	53	52
**Initiation of long distance oriented flight**	Bird ID<br>MinPress<br>Humidity<br>Rain_3Wks<br>Rain_Week	0.88	0.63	57	57
